# Pigment Epithelium-Derived Factor 34-mer Peptide Prevents Liver Fibrosis and Hepatic Stellate Cell Activation through Down-Regulation of the PDGF Receptor

**DOI:** 10.1371/journal.pone.0095443

**Published:** 2014-04-24

**Authors:** Tung-Han Tsai, Shou-Chuan Shih, Tsung-Chuan Ho, Hsin-I Ma, Ming-Ying Liu, Show-Li Chen, Yeou-Ping Tsao

**Affiliations:** 1 Department of Neurosurgery, Tri-Service General Hospital, National Defense Center, Taipei, Republic of China; 2 Department of Gastroenterology, Mackay Memorial Hospital, Taipai, Republic of China; 3 Mackay Medicine, Nursing and Management College, Taipei, Republic of China; 4 Department of Medical Research, Mackay Memorial Hospital, Taipei, Republic of China; 5 Department of Microbiology, School of Medicine, National Taiwan University, Taipei, Republic of China; National Cancer Center, Japan

## Abstract

Pigment epithelium-derived factor (PEDF) has been shown previously to prevent liver fibrosis and hepatic stellate cell (HSC) activation. By investigating the functional domains in PEDF, we identified a 34-mer peptide (residues Asp44-Asn77) that harbors the same function as the full-length PEDF protein. Not only did the 34-mer suppress the development of fibrosis in carbon tetrachloride (CCl_4_)-treated mouse liver but it also upregulated peroxisome proliferator-activated receptor-gamma (PPARγ) expression in HSCs *in vivo*. Platelet-derived growth factor (PDGF) plays a crucial role on the process of HSC activation in response to liver damage. The 34-mer suppressed PDGF-induced cell proliferation and expression of myofibroblastic marker proteins in primary rat HSC culture, increased the levels of PPARγ mRNA and protein in a dose-dependent manner and markedly reduced the level of active β-catenin protein, an HSC activating factor, in HSC-T6 cells. Similarly, IWR-1, an inhibitor of the Wnt response, displayed the same effect as the 34-mer in preventing HSC-T6 activation. The Wnt signaling-mediated PPARγ suppression was abolished by both the IWR-1 inhibitor and a small interfering RNA (siRNA) targeting β-catenin and the Wnt coreceptor, LRP6. Both PEDF and the 34-mer down-regulated PDGF receptor-α/β expression and blocked the PDGF-induced phosphorylation of Akt and ERK. Moreover, the inhibitory effect on PDGF receptor expression was abolished by PPARγ antagonists and PPARγ siRNA. Our observations indicate that the PEDF-derived 34-mer peptide can mimic PEDF in attenuating HSC activation. Investigation of this 34-mer peptide led to the identification of a signaling mechanism involving PPARγ induction, suppression of Wnt/β-catenin signaling and down-regulation of the PDGF receptor-α/β.

## Introduction

Hepatic stellate cells (HSCs) have proliferative potential, they are quiescent in the healthy liver and are activated by inflammatory stimuli that lead to the manifestation of a myofibroblast-like phenotype, including the expression of the myofibroblast marker, α-smooth muscle actin (α-SMA) [1]. Activated HSCs are the primary producers of extracellular matrix components during liver fibrogenesis [2]. Although liver fibrogenesis is a common intrinsic mechanism for wound healing [3], it also may be generated by a prolonged repair process in response to continued liver injury of various origins, ultimately leading to liver failure.

The platelet-derived growth factor (PDGF) family includes five dimeric proteins: PDGF-AA, -BB, -CC, –DD and PDGF-AB [4]. The expression of PDGF and its receptor subunits is limited to the mesenchymal cells of the portal tract stroma and vessels in the normal liver [5]. In the cirrhotic liver, the expression of PDGF-A and -B chains increases significantly in infiltrating inflammatory cells [5] and activated HSCs [6]. PDGF exerts its action by binding to two tyrosine kinase receptors (PDGFR-α and PDGFR-β), followed by receptor dimerization and signal transduction, following receptor auto-phosphorylation [4]. PDGFR-α binds to most PDGF dimers, except the DD-dimer, whereas the PDGFR-β binds to the BB- and DD-dimers [4]. Overexpressed PDGFR-α and –β in activated HSCs results in PDGF acting as a mitogen for myofibroblastic HSCs [7] and increases the number of fibrogenic cells accordingly, thereby promoting the development of liver fibrosis. Development of novel agents specifically targeting the PDGFR has been considered to be valuable in preventing hepatic fibrosis [8].

Pigment epithelium-derived factor (PEDF) is a 50 kDa secreted glycoprotein with multiple functions, including antiangiogenic effect on endothelial cells and neurotrophic activity on neurons. Two functional motifs have been identified within human PEDF, the 34-mer (amino acid positions Asp44-Asn77) and the 44-mer (Val78-Thr121), which are responsible for the antiangiogenic activity and neurotrophic activity, respectively [9], [10].

Recent studies have confirmed that PEDF is expressed in hepatocytes, however, its level is reduced dramatically in the livers of cirrhotic patients [11], [12]. In an animal model, liver fibrosis induced by various toxic chemicals (carbon tetrachloride or thioacetamide) is ameliorated by human PEDF, overexpressed using an adeno-associated viral vector (AAV) [11]. Human and rat PEDF share 80% identity and 90% homology at the protein level. The 34-mer is conserved between the human and rat with 86% identity and 93% homology. Furthermore, human PEDF exhibits cross-species activity and can suppresses the activation of activated rat HSCs in culture [11]. Taken together, these studies suggest that hepatic PEDF may play a role in preventing liver fibrosis. However, whether the antifibrotic activity of PEDF on activated HSCs could be achieved by a short PEDF functional motif (the 34-mer PEDF) remains unclear. Confirming the activity of the short functional antifibrotic PEDF peptide may be valuable because, if it could be used therapeutically, it may have favourable pharmacologic dynamics and low manufacturing costs.

## Materials and Methods

### Materials

Waymouth medium, fetal bovine serum (FBS) and trypsin were purchased from Invitrogen (Carlsbad, CA). 5-bromo-2′-deoxyuridine (BrdU), rosiglitazone, ciglitazone, Tween-20, Hoechst 33258 dye, and formalin were from Sigma-Aldrich (St. Louis, MO). Antibodies used in this study were for BrdU, α-SMA, cyclin D1, β-catenin, PDGFR-α and PDGFR-β (GeneTex, San Antonio, TX), desmin (Abcam, Cambridge, MA), p-ERK1/2 (Promega, Madison, WI),PPARγ, LRP6, phospho-LRP6 (Ser1490), non-phospho (Active) β-catenin (Ser33/37/Thr41), ERK1/2, Akt, and p-Akt (Cell Signaling Technology Inc., Beverly, MA), type I collagen 1A1 and β-actin (Santa Cruz Biotechnology Inc., Santa Cruz, CA). All fluorescent dye-conjugated secondary antibodies were purchased from BioLegend (San Diego, CA). GW9662 and G3335 were purchased from Calbiochem (La Jolla, CA). Human PDGF-BB and human Wnt3a were purchased from R&D Systems (Minneapolis, MN). Recombinant human PEDF derived from stable BHK cell transfectants and preserved in 50 mM sodium phosphate buffer (solvent) was obtained from Chemicon (Temecula, CA). PEDF peptides 18-mer (Glu97-Ser114), 34-mer and 44-mer were synthesized and modified with acetylation at the NH_2_ termini and amidation at the COOH termini for stability, and characterized by mass spectrometry (>95% purity) at GenScript (Piscataway, NJ). PEDF peptides were reconstituted in DMSO and used as stock solution (5 mM).

### Animal Treatment

Experimental procedures were approved by the Mackay Memorial Hospital Review Board and conducted according to national animal welfare regulations. To induce liver fibrosis, six-week-old female C57BL/6 mice (six mice per experimental condition) were injected intraperitoneally twice a week with CCl_4_ solution (5 ml/kg body weight as a 1∶4 mixture with olive oil) for 3 weeks. Subsequently, the 34-mer peptide and 18-mer control peptide at 10 mg/kg were administered by intraperitoneal injections twice a week and were continuously injected with CCl_4_ for a further 4 weeks.

### Cell isolation, culture, and treatment

Isolation and culture of primary rat HSCs were performed as in our previous study [11]. Briefly, primary rat HSCs were isolated by *in situ* portal vein perfusion with collagenase from the livers of male Sprague-Dawley rats (300–450 g). HSCs were then purified and enriched by Percoll density gradient centrifugation. Cells were cultured in Dulbecco's modified Eagle's medium (DMEM) supplemented with 20% fetal bovine serum (FBS) and 1% penicillin/streptomycin, and plated in 6 well culture dishes (Costar, Cambridge, Mass.). After plating for 24 h, non-adherent cells and debris were removed by washing with PBS and were then cultured in 10% FBS-DMEM for a further 6 days. Cell purity was verified as approximately 95∼98% by vitamin A fluorescence 2 days after isolation. Subsequently, the HSCs were incubated in 1% FBS-DMEM for 2 days with or without PEDF peptide and then used for experiments.

HSC-T6 cells were grown in Waymouth medium supplemented with 10% FBS at 37°C in a humidified atmosphere of 5% CO_2_ [13]. Treatments with 4.5 nM PEDF, an optimal dosage for inactivating HSC-T6 cell determined from our previous study [11], or PEDF-derived peptides (100 nM, unless specified otherwise), were performed after cells were transferred to 1% FBS medium.

### Sirius-Red Staining

Deparaffinized liver tissue sections were stained for 1 h in 0.1% (w/v) Sirius red (Sigma-Aldrich) in a saturated aqueous solution of picric acid, and then rinsed for 30 min in 0.01 N acetic acid to remove unbound dye. For semi-quantitative analysis of liver fibrosis, 10 fields from each slide were randomly selected under a light microscope and the red-stained area per total area (mm^2^/mm^2^) was measured using the Image-Pro Plus 4.5.1 system.

### BrdU labeling

1×10^5^ primary rat HSCs or HSC-T6 cells were seeded onto a FNC solution (Athena Enzyme Systems, Baltimore, MD, USA)-coated slide and incubated with 10% FBS medium for 1 day and exposed to 1% FBS medium supplemented with PEDF or PEDF peptide for 2 days. The cells were then exposed to fresh 1% FBS medium containing 20 ng/ml PDGF-BB for another day and then BrdU (final concentration, 10 µM) was added to the culture for 2 h. After fixing with 4% paraformaldehyde, the cells were treated with 1 N HCl at RT for 1 h and then exposed to cold methanol for 2 min prior to staining for immunofluorescence.

### Immunofluorescence

Deparaffinized tissue sections or 4% paraformaldehyde-fixed primary rat HSCs were blocked with 10% goat serum and 5% BSA in PBS containing 0.1% Tween-20 for 1 h. Staining was done using primary antibodies against α-SMA (1∶100 dilution), desmin (1∶100 dilution), PPARγ (1∶100 dilution) and BrdU (1∶100 dilution) at 37°C for 2 h, followed by incubation with the appropriate rhodamine- or FITC-conjugated donkey IgG (1∶500 dilution) for 1 h at room temperature (RT). Changes in F-actin structures were detected by 0.33 mM rhodamine-conjugated phalloidin (Sigma-Aldrich) for 1 h at RT. The cell numbers were monitored by counterstaining with Hoechst 33258 (Sigma-Aldrich) for 7 min. After final washes and mounting, average numbers of BrdU-positive cells were calculated in ten randomly selected fields of three different chambers (∼1×10^4^ cells). Images were captured using a Zeiss epifluorescence microscope with a CCD camera and photographs taken using the Axiovert software.

### RNA extraction and quantitative real-time PCR

Experiments were performed as previously described [14]. The sequences of the specific PCR primers were rat PPARγ(accession number: AF156665) sense, 5′- cccaatggttgctgattaca -3′, anti-sense, 5′- ggacgcaggctctactttga -3′; rat PDGFR-α(accession number: Z14118) sense, 5′- ctcccaggcctttctggt -3′, anti-sense, 5′- aagagctggcagacgatgag -3; rat PDGFR-β(accession number: Z14119) sense, 5′- gcggaagcgcatctatatct -3′, anti-sense, 5′- aatgaataggtcctcagagtccat -3; rat GAPDH (accession number: X02231) sense, 5′- tgggaagctggtcatcaac -3′, anti-sense, 5′- catcaccccatttgatcttga -3; mouse α-SMA (accession number: X13297) sense, 5′- atggctctgggctctgtaag -3′, anti-sense, 5′- tctgggacgtcccacgatgga -3; mouse COL1A1 (accession number: U38544) sense, 5′- cctcatttgcctttctctcc -3′, anti-sense, 5′- ctccaaagagacttactcgg -3; mouse TGFβ1(accession number: M13177) sense, 5′- tgcgcttgcagagattaaaa -3′, anti-sense, 5′- ctgccgtacaactccagtga -3; mouse PPARγ(accession number: U01841) sense, 5′- ccaagaataccaaagtgcga -3′, anti-sense, 5′- tagaaggttcttcatgggcc -3′; mouse PDGF-A(accession number: NM_008808) sense, 5′- cgaagtcagatccacagcat-3′, anti-sense, 5′- gggctctcagacttgtctcc-3′; mouse PDGF-B(accession number: NM_011057) sense, 5′- gcaccgaaagtttaagcaca -3′, anti-sense, 5′- aaataaccctgcccacactc -3′; mouse PDGF-C(accession number: NM_019971) sense, 5′- gaacagaacggagtgcaaga -3′, anti-sense, 5′- atctccacaccagcaccata -3′; mouse PDGF-D(accession number: NM_027924) sense, 5′- aggagctgaagctgaccaat -3′, anti-sense, 5′- tcactgtcttccctgagctg -3′; mouse PDGFR-α(accession number: NM_011058) sense, 5′- caaaccctgagaccacaatg -3′, anti-sense, 5′- taatggtgggaagggaagag -3′; mouse PDGFR-β(accession number: NM_008809) sense, 5′- agccagaagtagcgagaagc -3′, anti-sense, 5′- ggcagtattccgtgatgatg -3′; and mouse GAPDH (accession number: NM_008084) sense, 5′- acaactttggcattgtggaa -3′, anti-sense, 5′- gggcagccccacggccatca -3.

### Western blot analysis

Cells were scraped into lysis buffer (150 µL/35-mm well) containing 20 mM HEPES (pH 7.4), 1% SDS, 150 mM NaCl, 1 mM EGTA, 5 mM β-glycerophosphate, 10 mM sodium pyrophosphate, 10 mM sodium fluoride, 100 µM sodium orthovanadate, 10 µg/mL leupeptin, and 10 µg/mL aprotinin. The lysate was resolved by SDS-PAGE, electrotransferred to polyvinylidene difluoride membranes (Millipore, Bedford, MA), and processed for immunoblot analysis. Antibodies used in the immunoblot study included type I collagen 1A1, α-SMA, cyclin D1, ERK1/2, Akt, PPARγ,LRP6, phospho-LRP6, β-catenin, non-phospho (Active) β-catenin, PDGFR-αand PDGFR-β(1∶1000 dilution), p-ERK1/1 and p-Akt (1∶500 dilution), and β-actin (1∶5000 dilution). Proteins of interest were detected using the appropriate IgG-HRP secondary antibody and ECL reagent. X-ray films were scanned on a Model GS-700 Imaging Densitometer (Bio-Rad Laboratories, Hercules, CA) and analyzed using Labworks 4.0 software. For quantification, blots from at least three independent experiments were used.

### PPARγ small interfering RNA (siRNA) treatment

Rat LRP6 siRNA was purchased from Cell Signaling Technology (#9834; SignalSilence® LRP6 siRNA I). Rat specific β-catenin siRNA was purchased from Santa Cruz Biotechnology (sc-270011). A mixture of four rat PPARγ siRNAs (SMART-pools) and a species-specific siCONTROL non-targeting siRNA were purchased from Dharmacon Research (Lafayette, CO). For the transfection procedure, cells were grown to 60% confluence and siRNA was transfected using INTERFERin siRNA transfection reagent (PolyPlus-Transfection, San Marcos, CA) according to the manufacturer's instructions. The final concentration of siRNA was 10 nM. At 16 h after siRNA transfection, cells were resuspended in new media for a 24 h recovery period.

### Statistics

The results are expressed as the mean ± standard error of the mean (SEM). ANOVA was used for statistical comparisons. *P*<0.05 was considered significant.

## Results

### The 34-mer suppresses the development of fibrosis in CCl_4_-treated mouse livers

**Figure 1:**
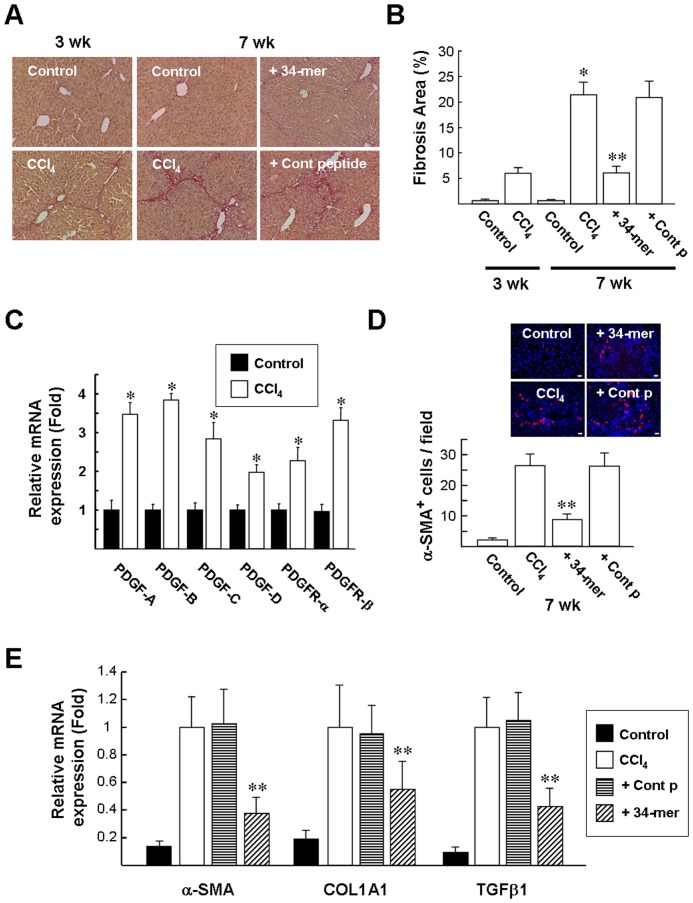
Suppression of CCl_4_-induced liver fibrosis by the 34-mer. Liver fibrosis was induced by intraperitoneal injection of CCl_4_ twice a week for 3 weeks. The mice then received the 34-mer twice a week intraperitoneally and were continuously injected with CCl_4_ for further 4 weeks. (**A**) Histopathological detection of collagen in the liver by Sirius red-staining. (Original magnification, ×200). Representative pictures from at least 3 different experiments with six mice in each subgroup are shown. (**B**) Estimation of hepatic fibrosis area by Sirius red staining. Data were assessed by analyzing 10 Sirius red-stained liver sections per animal with a computerized image-plus system. *P<0.02 *versus* CCl_4_-treated 3 weeks-group; ^**^ *P*<0.001 *versus* control peptide+CCl_4_-treated group. (**C**) Relative mRNA expression of PDGF isoforms and receptors in CCl_4_-treated mouse livers. CCl_4_ treatment was performed as described as above. The cycle threshold (*Ct*) value of the PCR product and a control mRNA (GAPDH) were used to calculate the relative quantities of mRNA between samples. Data represent means ± SD of 6 animals in each group. *P<0.05 versus untreated group. (D) Specimens were immunohistologically stained with α-SMA to observe activated HSCs (red color) and counterstained with Hoechst 33258 to observe nuclei. Original magnification, ×400. Scale bar  = 20 µM. Numbers of α-SMA-positive cells are expressed as α-SMA-positive cells/400× field. Results were evaluated from 6 sections per liver specimen, and 6 mice in each group. ^**^ *P*<0.005 *versus* control peptide+CCl_4_-treated group. (**E**) qPCR analysis. The mRNA expression of TGFβ1, CoL1A1 and α-SMA was significantly attenuated by the 34-mer. The cycle threshold (*Ct*) value of the PCR product and a control mRNA (GAPDH) were used to calculate relative quantities of mRNA between samples. Data represent means ± SD of 6 animals in each group. **P<0.05 versus control peptide+CCl_4_-treated group.

Using a CCl_4_-induced liver fibrosis mouse model, we investigated whether the 34-mer could attenuate hepatic fibrosis in vivo. Mice were administered CCl_4_ intraperitoneally and the area of liver fibrosis was quantified by Sirius red staining (Fig. 1A). CCl_4_ treatment for 3 weeks induced minimal fibrosis, whereas treatment for 7 weeks resulted in a significant increase in intrahepatic collagen content (6.0±1.1% *versus* 21.4±2.4%; Fig. 1B). In addition, after CCl_4_ treatment twice a week for 3 weeks, relative mRNA levels of the PDGF isoforms and PDGFR-α/β in the mouse livers were evaluated by quantitative real-time RT-PCR (qPCR) and the results revealed that CCl_4_ treatment increased these mRNA levels by ∼2–4 fold compared to the untreated control (Fig. 1C). To investigate the therapeutic effect of PEDF peptides, animals were randomly assigned into two groups after CCl_4_ treatment for 3 weeks and treated with the 34-mer or the control peptide (an 18-mer PEDF peptide) by intraperitoneal injection twice a week for 4 weeks. In addition, the mice were continuously injected with CCl_4_ for another 4 weeks. The animals were euthanized at the end of experiment and Sirius red staining indicated that treatment with the 34-mer significantly reduced the fibrotic area, compared to treatment with the control peptide (6.1±1.3% *versus* 20.9±3.1%; Fig. 1B).

Next, liver sections were stained for the activated HSC marker, α-SMA (400× field; Fig. 1D). Numerous α-SMA-positive cells, concentrated around portal areas, were identified in the CCl_4_–treated mice and CCl_4_ plus control peptide-treated mice, whereas the number of α-SMA-positive HSCs reduced dramatically in the presence of the 34-mer (26.3±3.6 and 26.2±4.5 versus 8.7±1.9). The expression of mRNA encoding α-SMA, COL1A1 and TGFβ1 was evaluated by qPCR and the results revealed that these transcripts were significantly increased at week 7 after CCl_4_ treatment (Fig. 1E). Specifically, the 34-mer diminished α-SMA, COL1A1 and TGFβ1 mRNA levels by a factor of 2.7-fold, 1.7-fold and 2.5-fold, respectively, compared to the levels in the control peptide group. We also evaluated the levels of α-SMA and COL1A1 in liver protein extracts harvested at week 7 (Fig. S1), in which the 34-mer diminished α-SMA and COL1A1 levels by a factor of 3.4-fold and 2.8-fold, respectively, compared to the levels in the control peptide group. Taken together, these data suggest that the 34-mer inhibits the activation of HSCs in a mouse model of CCl_4_-induced liver fibrosis.

### The 34-mer upregulates PPARγ expression in activated HSCs *in vivo*

**Figure 2:**
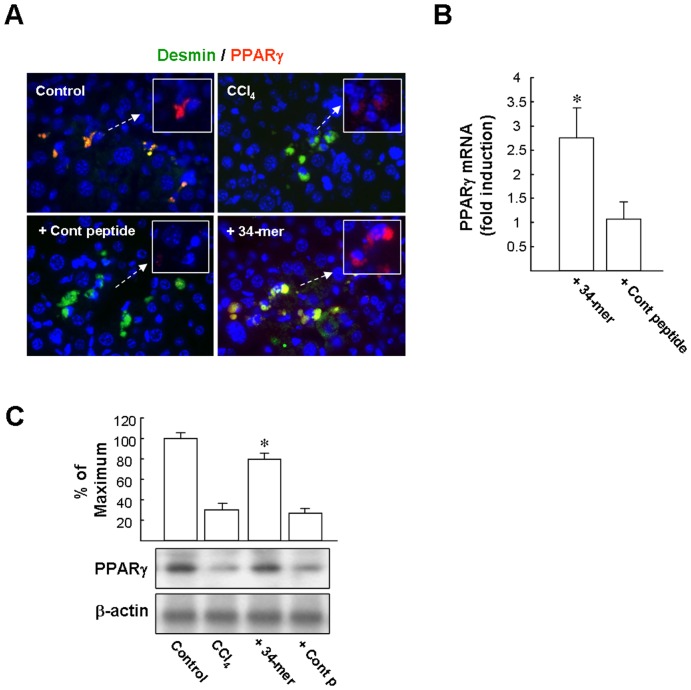
The 34-mer induces PPARγ expression in activated HSCs in vivo. Liver fibrosis was induced by intraperitoneal injection of CCl_4_ twice a week for 3 weeks. The mice were then injected with PEDF peptides every two days for a week. (**A**) Representative pictures of four independent experiments show dual-immunofluorescence staining of HSCs by desmin (green labeling), PPARγ (red labeling), and merged (*yellow*; PPARγ-positive HSCs). Original magnification, ×400. (**B**) PPARγ mRNA in liver extract assayed by qPCR. Relative mRNA expression levels were normalized to the GAPDH mRNA content. Data represent three independent experiments and six mice per group. *P<0.001 versus control peptide+CCl_4_-treated group. (**C**) Liver protein extracts were harvested and subjected to western blot analysis with antibodies as indicated. Representative blots and densitometric analysis from 3 independent experiments are shown. *P<0.05 versus control peptide+CCl_4_-treated group.

To explore the protective effect of the 34-mer on liver fibrogenesis, we investigated whether the 34-mer could induce PPARγ expression in activated HSCs *in vivo*. To activate HSCs, mice were injected with CCl_4_ twice a week for 3 weeks. Subsequently, the mice were injected intraperitoneally with PEDF peptides (34-mer or 18-mer) every 2 days for a week. Liver sections were examined by dual-immunofluorescence staining for PPARγ (red) and the HSC marker desmin (green). As depicted in Fig. 2A, PPARγ labeling was detected in desmin-positive HSCs in the liver tissue of the untreated controls. CCl_4_ treatment increased the number of HSCs, however, only a few HSCs stained positively for PPARγ (faint red). 34-mer treatment partially recovered PPARγ expression in these desmin-positive HSCs. qPCR analysis revealed that 34-mer treatment increased the PPARγ mRNA levels by about 2.8±0.6-fold compared to that of the control peptide-treated group (Fig. 2B). Western blotting analysis revealed a dramatic reduction in PPARγ protein level in the livers after administering CCl_4_ for 3 weeks (Fig. 2C). The decline in PPARγ protein level was significantly reversed (about 80%) after 34-mer treatment for 1 week. In contrast, the control peptide had no such effect.

### PEDF and the 34-mer reverse HSC activation induced by PDGF

**Figure 3:**
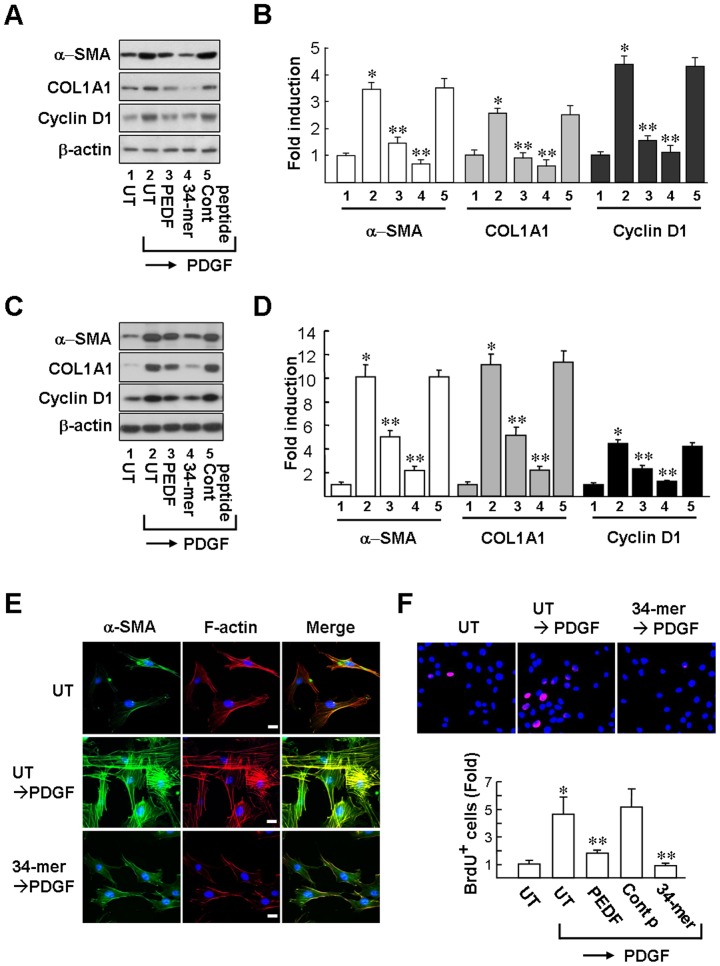
PEDF and the 34-mer inhibit HSC activation induced by PDGF. HSC-T6 cells (**A and B**) and primary rat HSCs (**C and D**) were either untreated or pretreated with PEDF or the 34-mer for 2 days and then stimulated by PDGF (20 ng/ml) for 24 h. Representative western blots (**A and C**) and densitometric analysis with SD (**B and D**) from 3∼4 separate experiments are shown. *P<0.05 versus UT cells (column 1). **P<0.05 versus control peptide/PDGF-treated cells (column 5). (**E**) Immunofluorescence analysis. F-actin (*red*) and α-SMA (*green*) in primary rat HSCs were stained by rhodamine-conjugated phalloidin and anti-α-SMA antibody, respectively. DNA was visualized with Hoechst 33258 staining. Scale bar: 20 µM. Original magnification ×400. Representative images from three independent experiments. (**F**) BrdU labeling assay. BrdU-positive cells (*red*) were detected by immunofluorescence microscopy (original magnification, ×400). Variations shown represent SD from three independent experiments. *P<0.01 versus UT cells. **P<0.05 versus control peptide/PDGF-treated cells.

To investigate whether the PEDF-derived short peptide retains the full length PEDF effect of inactivating HSCs, *in vitro* studies were conducted using immortalized rat HSCs (HSC-T6 cell) and primary rat HSCs. HSCs were pretreated with PEDF or PEDF-derived peptides (34-mer and 44-mer) for two days, followed by PDGF stimulation for one day. As shown in Fig. 3, PDGF stimulation induced HSC activation, in which increased cell proliferation and fibrogenic responses were observed. Exposure of HSC-T6 cells to PDGF led to a 3.5±0.24-fold and 2.6±0.18-fold induction of the α-SMA and COL1A1 proteins, respectively, compared to UT cells (Figs. 3A and 3B). Likewise, exposure of primary rat HSCs to PDGF increased the α-SMA and COL1A1 proteins by 10.1±1.0-fold and 11.1±0.91-fold, respectively, compared to UT cells (Figs. 3C and 3D). Importantly, this PDGF induced α-SMA and COL1A1 expression was markedly reduced by pretreatment with PEDF or the 34-mer; whereas pretreatment with the 44-mer did not give such an effect in primary rat HSCs or HSC-T6 cells.

We further examined stress fiber architecture inside HSCs, a characteristic feature of HSC activation induced by PDGF [4], by rhodamine-phalloidin staining of filamentous (F-) actin and immunofluorescence staining of α-SMA. As depicted in Fig. 3E, abundant staining of F-actin and α-SMA were observed in the cytoplasm of PDGF-treated primary rat HSCs, compared to untreated cells. In contrast, PDGF exerted no such effect on 34-mer-pretreated cells, indicating that the activation of HSCs by PDGF is abolished by the 34-mer.

PDGF can also induce HSC proliferation, as evident from the increase of cyclin D1, a key regulator of transition from G1 into the S phase of the cell cycle. PEDF and the 34-mer also reduced PDGF-induced cyclin D1 protein levels (Figs. 3A–3D). This finding suggested that both PEDF and the 34-mer may suppress HSC proliferation induced by PDGF. HSC proliferation was also investigated by following DNA synthesis with BrdU pulse-labeling assay. Approximately 3% BrdU-positive primary rat HSCs were identified in untreated (UT) cells. Exposure of the UT cells to PDGF increased the BrdU-positive ratio by 4.6±1.2-fold (UT/PDGF; Fig. 3F). However, exposure of the PEDF- and 34-mer-treated cells to PDGF increased the BrdU-positive cells by only 1.8±0.19-fold and 0.88±0.17-fold, respectively. This indicates that the mitogenic activity of PDGF is suppressed by pretreatment with PEDF or the 34-mer. Pretreatment with the 44-mer control peptide, on the other hand, failed to suppress PDGF-induced cell proliferation. A trypan blue exclusion test was used to exclude the possibility that the reduction of cell proliferation was attributable to a cytotoxic effect of PEDF/34-mer on primary rat HSCs (data not shown). Collectively, PEDF and its 34-mer peptide can prevent PDGF-induced activation and proliferation of primary rat HSCs and HSC-T6 cells.

### The 34-mer upregulates PPARγ expression and reduces β-catenin protein in HSC-T6 cells

**Figure 4:**
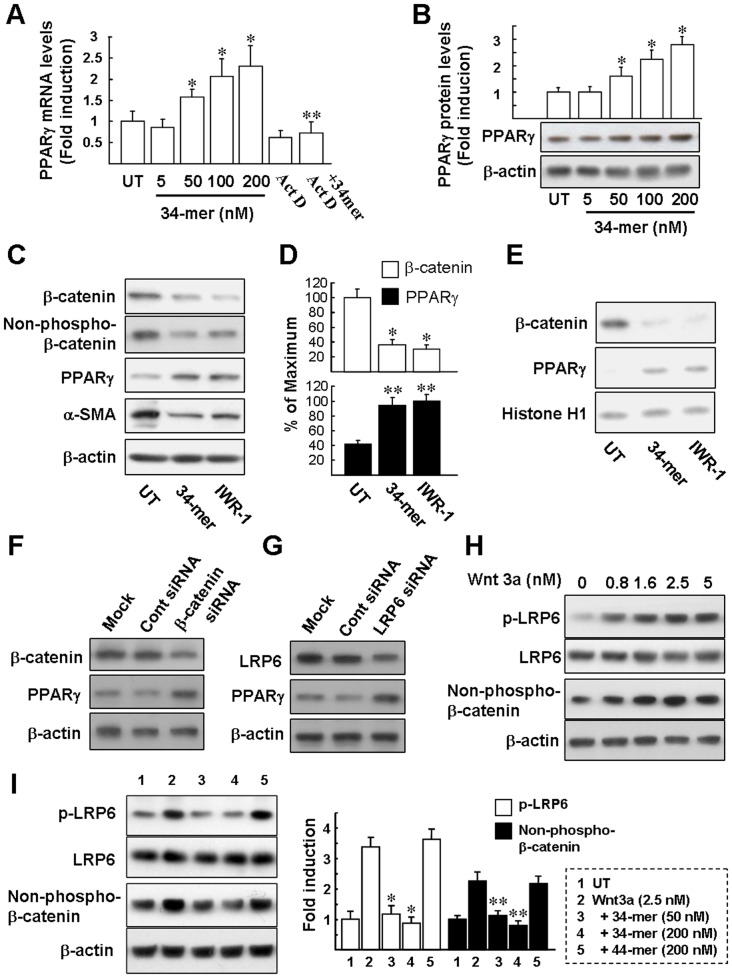
The34-mer induces PPARγ expression via suppression of Wnt signaling. (**A**) qPCR. Cells were treated with various doses of the 34-mer for 24 h or pretreated with 5 ng/ml actinomycin D (Act D) for 1 h and then incubated with the 34-mer for an additional 24 h. Average levels of PPARγ mRNA from three repeats of experiments are shown. *P<0.005 versus UT cells. **P<0.005 versus 100 nM 34-mer-treated cells. (B) HSC-T6 cells were treated with the 34-mer at the indicated concentrations for 48 h and proteins were detected by western blot analysis with the antibodies indicated. Representative blots (below panels) and densitometric analysis with SD (upper figures) of three independent experiments are shown. *P<0.05 versus UT cells. The 34-mer suppresses β-catenin protein expression. HSC-T6 cells were exposed to the 34-mer or IWR-1 for 48 h and then total cell lysates were prepared for western blotting using the antibodies indicated. Representative blots (**C**) and densitometric analyses with SD (**D**) from four separate experiments are shown. *P<0.01 versus untreated cells; **P<0.05 versus untreated cells; (**E**) HSC-T6 cells were exposed to the 34-mer or IWR-1 for 48 h and then nuclear extracts were prepared and subjected to western blot analysis of nuclear β-catenin and PPARγ. Relative nuclear protein expression levels were normalized to histone H1. Representative blots from two independent experiments. (**F and G**) Suppression of Wnt signaling by β-catenin siRNA or LRP6 siRNA upregulates PPARγ expression in HSC-T6 cells. The cells were transfected with β-catenin, LRP6 siRNA or control siRNA for 16 h, allowed to recover in complete medium for a further 48 h and then were harvested for western blot analysis. “Mock” indicates that the cells were treated with transfection reagent alone. Representative blots from three independent experiments. (**H**) HSC-T6 cells were treated with Wnt3a at the indicated concentrations for 1 h and proteins were detected by western blot analysis with antibodies against the active phosphorylated form of LRP6 and active β-catenin. Equal protein loading was confirmed by reprobing the membranes with anti-LRP6 or β-actin antibodies. Representative blots from three separate experiments are shown. (**I**) The 34-mer suppresses Wnt3a-induced LRP6 phosphorylation and active β-catenin formation. HSC-T6 cells were treated with Wnt3a or co-treated with Wnt3a and the 34-mer/44-mer at the indicated concentrations for 1 h and then proteins were detected by western blot analysis with the antibodies indicated. Representative blots (left panels) and densitometric analysis with SD (right figures) of three independent experiments are shown. *P<0.005 versus Wnt3a-treated cells; **P<0.01 versus Wnt3a-treated cells.

The ability of 34-mer to induce PPARγ gene expression in HSC-T6 cells was investigated. After stimulation with 50–200 nM of the 34-mer for 24 h, qPCR analysis revealed that PPARγ mRNA levels had increased in a dose-dependent manner (1.6±0.19-fold to 2.3±0.49-fold; Fig. 4A). Furthermore, the 34-mer-mediated induction of transcription was completely blocked by actinomycin D pretreatment, suggesting that the increase in mRNA concentrations is transcription dependent. After stimulation 48 h, the 34-mer also increased PPARγ protein levels in a dose-dependent manner, assayed by western blotting (1.6±0.37-fold to 2.8±0.30-fold; Fig. 4B).

Wnt/β-catenin signaling is associated with maintenance of HSC activation by a mechanism involving the down-regulation of PPARγ expression [15], [16]. One conceivable mechanism by which the 34-mer induces PPARγ expression may be through inhibition of Wnt/β-catenin. Western blot analysis revealed that 34-mer treatment significantly reduced the expression of total and active β-catenin protein in HSC-T6 cells, while the PPARγ protein level increased by about 2.3-fold (Figs. 4C and 4D). A similar effect was achieved using the Wnt antagonist IWR-1 (Figs. 4C and 4D). Reduction of α-SMA protein levels following 34-mer or IWR-1 treatment supported the conclusion that HSC-T6 cell activation was suppressed. As illustrated by the immunoblot in Fig. 4E, 34-mer or IWR-1 treatment caused a decrease in nuclear β-catenin levels, suggesting that β-catenin-mediated transcription is suppressed. To investigate the specific effect of β-catenin on PPARγ expression, HSC-T6 cells were transfected with a β-catenin-specific siRNA. Western blotting verified the function of the siRNA, in that the protein level of β-catenin was significantly reduced (Fig. 4F, blot 1). Importantly, β-catenin siRNA treatment significantly increased PPARγ protein expression compared to either mock or control siRNA transfection (blot 2). These results confirmed the importance of β-catenin in repressing PPARγ expression in HSCs.

Wnt-induced LRP6-Frizzled receptor dimerization is an essential step in canonical Wnt signaling, promoting LRP6 phosphorylation to initiate β-catenin-mediated signaling [17]. This raises the possibility that LRP6 plays a role in suppressing PPARγ expression in HSCs. To address this, we analyzed the effect of LRP6 siRNA on PPARγ derepression in HSC-T6 cells. As shown in Fig. 4G, siRNA transfection caused a marked reduction in LPR6 protein expression and increased PPARγ expression compared to either mock or control siRNA transfection. This suggests that LRP6 participates in Wnt-mediated PPARγ suppression in HSCs. Taken together, these results suggest that the blockade of Wnt signaling may represent an important molecular event during PPARγ induction in HSCs. We investigated whether Wnt3a affects Wnt signaling in HSC-T6 cells by western blot analysis using antibodies against the active phosphorylated form of LRP6 (p-LRP6) and active β-catenin. After stimulation with 0.8-5 nM of Wnt3a for 1 h, p-LRP6 levels increased in a dose-dependent manner (1.5±0.14-fold to 3.6±0.31-fold; Fig. 4H). Meanwhile, Wnt3a also increased active β-catenin levels in a dose-dependent manner, assayed by western blotting (1.7±0.26-fold to 2.4±0.27-fold). We next analyzed whether 34-mer could prevent the Wnt3a-induced LRP6 phosphorylation. HSC-T6 cells were cotreated with both 34-mer (50 nM or 200 nM) and Wnt3a (2.5 nM) for 1 h and western blot analysis revealed that the 34-mer almost completely abrogated the Wnt3a-induced LRP6 phosphorylation and active β-catenin formation. The 44-mer control peptide had no such effects. Collectively, these results indicate that the 34-mer suppresses Wnt/β-catenin signaling by a mechanism involving interference with Wnt-induced LRP6 phosphorylation.

### PEDF and the 34-mer down-regulate PDGFR expression in HSC-T6 cells

**Figure 5:**
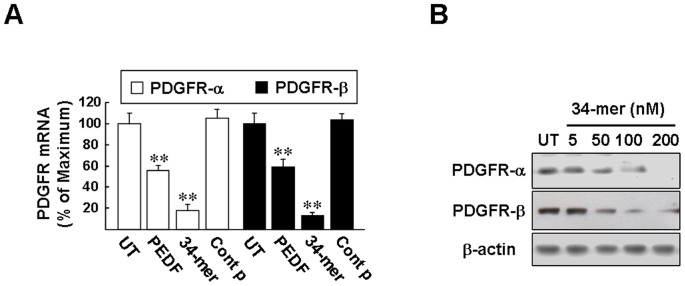
PEDF and the 34-mer suppress PDGFR expression in HSC-T6 cells. (**A**) qPCR. HSC-T6 cells were cultured in 1% FBS medium for 2 days (UT) or 1% FBS medium supplemented with PEDF, the 34-mer or 44-mer for 2 days before RNA extraction. The cycle threshold (*Ct*) value of the PDGFR-α and –β mRNA and a control GAPDH mRNA were used to calculate relative quantities of mRNA between samples. Average levels of PDGFR-α and –β mRNA from three repeats of experiments are shown. **P<0.002 versus the 44-mer control peptide-treated cells. (**B**) HSC-T6 cells were treated with the 34-mer at the indicated concentrations for 48 h and proteins were detected by western blot analysis with the antibodies indicated. Representative blots from three independent experiments are shown.

HSC activation in response to liver injury is a progressive process and expression of PDGF receptors in HSCs is one of early events required for HSC expansion [18]. As illustrated in Fig. 5A, qPCR analysis showed that HSC-T6 cells expressed a basal level of PDGFR-α and –β mRNA (set as 100%) and, following exposure to PEDF and the 34-mer for 48 h, the levels of PDGFR-α mRNA markedly reduced to 55.5±4.94% and 18.0±5.49%, respectively, compared to that of the control. Similarly, PEDF and the 34-mer suppressed the levels of PDGFR-β mRNA to 59.0±7.47% and 13.3±2.66%, respectively. In contrast, the 44-mer control peptide had no such effect. Western blotting results revealed that exposure of HSC-T6 cells to 50–200 nM 34-mer for 48 h reduced PDGFR-α and –β protein levels in a dose-dependent manner (Fig. 5B), 100 nM 34-mer being the effective dosage for suppressing PDGFR-α and –β protein expression.

### PEDF suppresses PDGF-induced phosphorylations of ERK and Akt

**Figure 6:**
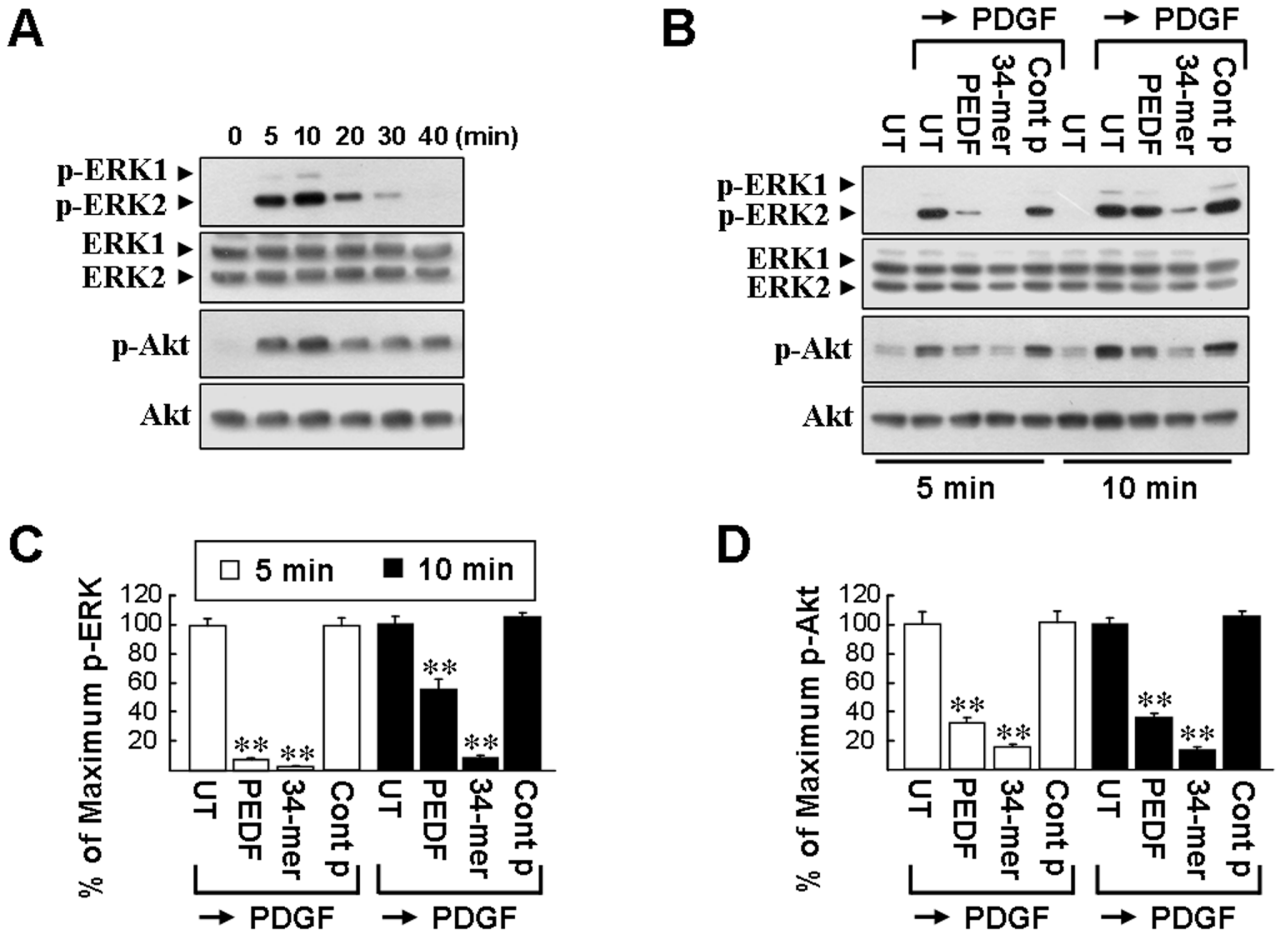
PEDF and 34-mer pretreatment suppress PDGF signaling. (**A**) PDGF induces ERK and Akt phosphorylation in HSC-T6 cells. Cells were cultured in 1% FBS medium for 2 days and then exposed to fresh serum-free medium containing PDGF for the indicated time periods. Western blotting was performed to detect the active phosphorylated forms of ERK (p-ERK) and Akt (p-Akt) and is shown in the upper panels. Equal protein loading was confirmed by reprobing the membranes with total ERK and Akt antibodies as a loading control. Representative blots from three separate experiments are shown. (**B**) Treatment of HSC-T6 cells with PEDF or the 34-mer prevents ERK and Akt phosphorylation induced by PDGF. HSC-T6 cells were untreated or pretreated with PEDF or the 34-mer for 2 days and then stimulated with PDGF for the indicated time periods. Cells were harvested and subjected to western blot analysis with phosphospecific antibodies to ERK1/2 and Akt. (**C and D**) After densitometric scanning of triplicate blots, values for p-ERK2 and p-Akt were normalized to total ERK2 and Akt, respectively. **P<0.05 versus control peptide→PDGF-treated cells.

PDGF-induced phosphorylation of ERK and Akt are regarded as essential steps leading to mitogenesis in HSCs [6], [19]. As depicted in Fig. 6A, phosphorylated ERK2 (p-ERK2; MW 42 kDa) and p-Akt appeared 5 min after HSC-T6 cells were treated with PDGF for intervals ranging between 5 and 40 min. The peak phosphorylation of ERK2 and Akt occurred between 5 and 10 min. There were no obvious changes in total ERK1/2 or Akt.

The levels of PDGF-induced p-ERK and p-Akt in PEDF- and 34-mer-treated cells were determined by western blotting (Fig. 6B). PEDF or 34-mer pretreatment markedly prevented the induction of p-ERK2 and p-Akt. Pretreatment with the 44-mer resulted in no significant effects on the levels of PDGF-induced p-ERK2 and p-Akt in any of the time periods studied. Statistically, PEDF and the 34-mer partially suppressed the ERK phosphorylation stimulated by PDGF for either 5 min (7.8±1.3% and 2.3±1.0%) or 10 min (57.1±4.9% and 7.8±1.6%), compared to that of the control cells (set as 100%; Fig. 6C). Also, PEDF and the 34-mer partially blocked PDGF-induced Akt phosphorylation for either 5 min (32.0±3.4% and 15.5±1.9%) or 10 min (35.8±2.6% and 13.3±1.9%), compared to the control cells (Fig. 6D).

### PPARγ mediates the suppressive effect of PEDF on PDGFR expression in HSCs

**Figure 7:**
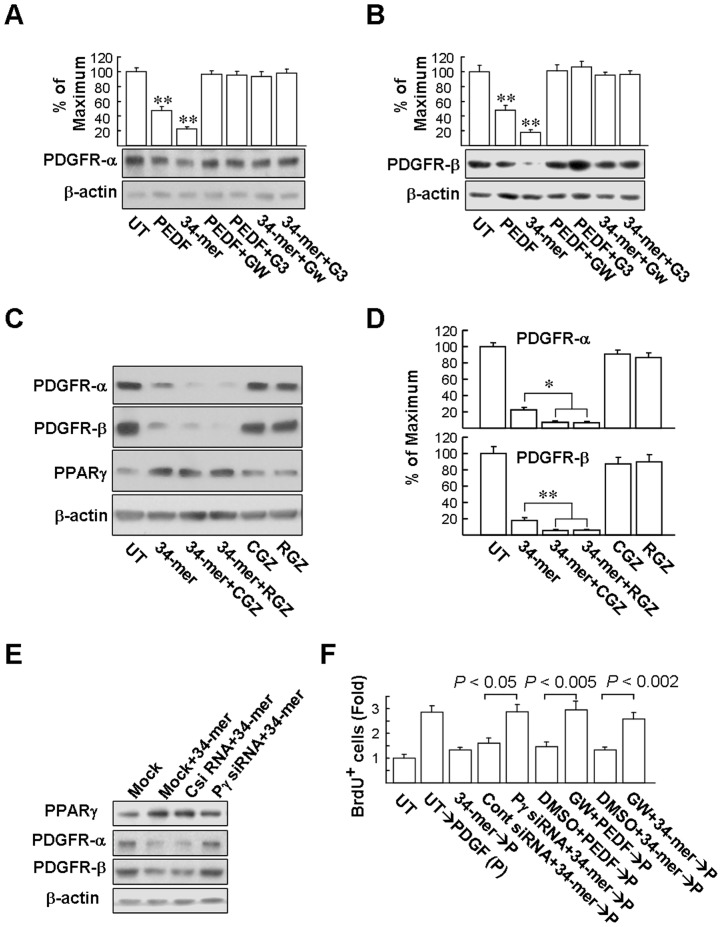
PEDF suppresses PDGFR expression by PPARγ. (**A** and **B**) PPARγ antagonists suppress the inhibitory effect of PEDF on PDGFR-α and –β expression. HSCT-6 cells were treated with PEDF or the 34-mer in combination with 10 µM GW9662 or 10 µM G3335 for 48 h. Cells were harvested for western blot analysis. Equal loading was confirmed with antibodies against β-actin. Representative blots (below panels) and densitometric analysis with SD (upper figures) of three independent experiments are shown. **P<0.0001 versus untreated cells. (**C** and **D**) The effect of synthetic PPARγ ligands CGZ and RGZ on PDGFR-α/β expression. HSCT-6 cells were treated with the 34-mer, 5 µM PPARγ ligand or the 34-mer combined with CGZ or RGZ for 48 h. Cells were harvested for western blot analysis. Equal loading was confirmed with antibodies against β-actin. Representative blots (C) and densitometric analysis with SD (D) of three independent experiments are shown. *P<0.05 versus 34-mer-treated cells. **P<0.02 versus 34-mer-treated cells. (**E**) PPARγ siRNA abrogates PDGFR down-regulation induced by the 34-mer. HSC-T6 cells were transfected with a PPARγ siRNA or control siRNA for 16 h and allowed to recover in complete medium for a further 24 h. “Mock” indicates that cells were treated with transfection reagents alone. After treatment, the HSC-T6 and siRNA-transfected HSC-T6 cells were exposed to the 34-mer for 48 h and then harvested for western blot analysis. (**F**) BrdU pulse-labeling assay. GW9662 and siRNA pretreatment was performed as described above, followed by PDGF (P) treatment for an additional 24 h and BrdU pulse-labeling for 2 h. Variations shown represent SD from three independent experiments (n = 3 dishes).

As depicted in Figs. 7A and 7B, PEDF suppressed PDGFR-α and –β protein expression to 47.5±5.07% and 48.0±6.39%, respectively. Moreover, 100 nM 34-mer could effectively suppress the expression of PDGFR-α and –β protein to 22.5±3.12% and 17.8±3.64%, respectively. PPARγ is a transcription factor that is activated by a variety of endogenous ligands, including fatty acids and eicosanoids [20]. We investigated whether the PPARγ antagonists GW9662 or G3335 could inhibit the effect of PEDF or the 34-mer on the down regulation of PDGFR. Western blot analysis revealed that PEDF/34-mer and PPARγ antagonist (10 µM, 48 h) co-treatment abolished the ability of PEDF and the 34-mer to suppress PDGFR-α and –β protein accumulation in HSC-T6 cells (Figs. 7A and 4B). Moreover, the suppressive effect of the 34-mer on PDGF-induced phosphorylation of ERK and Akt was abolished by co-treatment with the PPARγ antagonist (Fig. S2). These results suggest that PEDF/34-mer acts through endogenous ligand-dependent PPARγ activation to down-regulate PDGFR expression in HSC-T6 cells.

Next, we tested whether synthetic PPARγ ligands (ciglitazone, CGZ and rosiglitazone, RGZ) could suppress PDGFR expression in HSC-T6 cells. As shown in Fig. 7C, the 34-mer effectively suppressed the expression of PDGFR-α and –β protein, as described above. Interestingly, the inhibitory effect of the 34-mer on PDGFR-α expression was markedly enhanced when the cells were co-treated with either CGZ or RGZ (7.0±1.6% and 6.8±1.4% versus 22.5±3.1%). Likewise, CGZ and RGZ could markedly enhance the suppression of PDGFR-β induced by the 34-mer (5.6±1.0% and 5.8±0.9% versus 17.8.5±3.6%). On the other hand, CGZ and RGZ had no effect on 34-mer-mediated PPARγ upregulation or PPARγ basal level expression in HSC-T6 cells (Fig. 7C, blot 3). Notably, the western blot analysis indicated that CGZ and RGZ treatment alone resulted in slight down-regulation of PDGFR, compared to untreated control cells, but there is less significance (Fig. 7D). These results suggest that a critical level of PPARγ is required to prevent the PDGFR expression.

We then investigated the possible impact of 34-mer-induced PPARγ on the down-regulation of PDGFR. Transfection of a PPARγ siRNA into HSC-T6 cells substantially reduced the ability of the 34-mer to induce the production of PPARγ protein and suppress the accumulation of PDGFR-α and –β proteins in HSC-T6 cells, compared to cells transfected with a control siRNA (Fig. 7E). These findings indicate that the 34-mer reduced PDGFR expression in HSC-T6 cells by way of PPARγ.

The signaling role of PPARγ for the PEDF/34-mer-mediated inhibitory effect on PDGF-induced cell proliferation was also evaluated. A BrdU pulse-labeling assay revealed that the ability of the 34-mer to suppress PDGF-induced cell proliferation was significantly hampered in HSC-T6 cells pretreated with PPARγ-specific siRNA, compared to those transfected with a control siRNA (Fig. 7F). Furthermore, the mitogenic activity of PDGF was not inhibited in HSC-T6 cells treated with either **PEDF- or the 34-mer- in the presence of GW9662 (** **Fig. 7F** **)**.

Collectively, the data revealed that PEDF acts through its 34-mer motif to induce PPARγ. The PEDF-induced PPARγ in turn causes PDGFR down-regulation and thereby blocks HSC activation induced by PDGF-PDGFR signaling.

## Discussion

Previously, we reported that PEDF synthesis decreased dramatically in livers with fibrosis induced by CCl_4_, whereas over-expressing PEDF via a viral vector halted the progression of liver fibrosis in the experimental animal [11]. These findings support the proposition that PEDF acts as an intrinsic protective factor against liver cirrhosis. Although the concept has been proved, prevention of liver cirrhosis by *in situ* expression of PEDF via a viral vector seems impractical. We, therefore, have addressed this problem using another approach, by testing the PEDF activity of smaller fragments, rather than the full length protein. Accordingly, a short PEDF peptide, 34 amino acids in length and with the activity of the full length PEDF, has been identified. The 34-mer PEDF can be injected directly into the peritoneal cavity to efficiently ameliorate CCl_4_-induced liver fibrosis in mice and induces PPARγ expression in activated HSCs. By identifying this short injectable peptide, we have advanced the potential application of PEDF for liver cirrhosis therapy. Furthermore, new molecular targets of PEDF also have been identified in the present study. Specifically, we found that PEDF/34-mer treatment initiates a previously unidentified pathway to suppress HSC activation. This involves suppressing Wnt/β-catenin signaling and the down-regulation of PDGF receptor-α/β. The elucidation of the molecular mechanisms by which hepatic PEDF, an intrinsic anti-fibrotic factor, modulates PDGF receptor signaling in HSCs significantly extends our understanding of the development of liver fibrosis.

PPARγ is highly expressed in quiescent HSCs in the normal liver and its expression decreases dramatically in activated HSCs both *in vitro* [21], [22] and *in vivo* [22], [23]. In culture-activated HSCs, the expression of PPARγ via an adenoviral vector suppresses cell proliferation and the expression of fibrogenic genes such as type 1 collagen, α-SMA, and TGFβ1 [21]. Therefore, PPARγ may be able to return the activated HSCs to a less active state. Our previous in vitro study revealed that PEDF induces PPARγ expression in both HSC-T6 cells and culture-activated rat HSCs [11]. In this study, we found the 34-mer may restore the expression of PPARγ protein in HSC-T6 cells and activate HSCs *in vivo*. In addition, PPARγ is apparently the major mediator responsible for 34-mer induced HSC inactivation. This is based on our observation that the inhibitory effects of the 34-mer on PDGFR expression and PDGF-induced HSC-T6 proliferation are significantly attenuated by pretreatment of the cells with a PPARγ antagonist or specific siRNA. Not only are the results of this study in line with our previous finding but they also support the proposition that PEDF may protect individuals from liver cirrhosis by maintaining PPARγ levels in HSCs.

The molecular events associated with PPARγ down-regulation in activated HSCs remain mostly unknown. A recent study indicated that the elements of the Wnt pathway, including Wnt ligands, Frizzled receptors and LRP6 co-receptor, gradually increased during the process of activating HSCs in culture [15]. Moreover, the formation of Wnt autocrine triggers nuclear accumulation of β-catenin in culture-activated HSCs and this has been shown to be associated with PPARγ suppression [15], [16]. These previous findings suggest that PPARγ is negatively associated with Wnt signaling. In this study, we showed also that the nuclear β-catenin level was reduced before the PPARγ level started to increase in HSC-T6 cells. This suggests a possible inhibition of Wnt signaling that enables PPARγ induction. Interestingly, it has been demonstrated that PEDF can effectively block the Wnt-induced nuclear translocation of β-catenin in ARPE-19 cells [24]. Moreover, binding of PEDF to recombinant LRP6 has been demonstrated by a co-precipitation assay that leads to the blockade of Wnt-induced LRP6-Frizzled receptor dimerization, an essential step in canonical Wnt signaling [24]. Our findings suggest that the 34-mer peptide may also undergo such physical interaction with LRP6.

PDGF signaling via the PDGF receptors (*pdgfr-α and-β*) is of great importance in ECM production through HSCs and expansion of activated HSCs in the early stage of CCl_4_-induced liver fibrosis [18], [25]. Our study revealed that the 34-mer suppresses the expression of the PDGF receptors in HSC-T6 cells. Moreover, pretreatment of the cells with PPARγ siRNA eliminates the inhibitory effect. These results collectively indicate that upregulation of PPARγ by PEDF/34-mer is essential for interrupting the expression of PDGF receptors and PDGF-mediated post-receptor signaling in HSCs. Because HSCs are activated by PDGF, the coupling of PDGF receptor down-regulation and the prevention of PDGF-induced HSC activation strongly support the notion that PEDF abolishes the PDGF effect by attenuating its receptors. Furthermore, pretreatment with PEDF/34-mer for 48 h is required for significant reduction of the levels of *pdgfr* gene expression. It is reasonable to hypothesize that the time requirement is associated with PPARγ accumulation and turnover of preexisted PDGFR. PPARγ also has been reported to be critical for the induction of expression of glutamate-cysteine ligase (GCL), which leads to enhanced antioxidant glutathione (GSH) accumulation and suppressed *pdgfr-β* expression mediated by oxidative stress in activated HSCs [26]. Although similar PPARγ activation and *pdgfr-α/β* suppression occurred in response to PEDF, the involvement of GCL and GSH in PEDF-induced HSC inactivation remains unclear and awaits further investigation.

To gain comprehensive knowledge of the antifibrotic function of the 34-mer, further *in vivo* and *in vitro* studies are required on liver cells other than HSCs, such as hepatocytes, Kupffer cells and sinusoidal endothelial cells (SECs). Previous studies have demonstrated that 34-mer domain exerts the antiangiogenic activity of PEDF [10], [27] and suppresses angiogenesis in animals with choroidal neovascularization [27] or PC-3 prostate adenocarcinomas [10]. The SECs are anatomically co-localized with the HSCs in the hepatic sinusoids [28]. In the process of long-term liver fibrogenesis, angiogenesis by SECs enhances the survival of both pre-neoplastic hepatocytes and activated HSCs [28], [29]. This suggests that elimination of both the proliferating SECs and the activated HSCs may result in a more effective blockade of hepatic fibrosis. Our current data cannot rule out an indirect effect of the 34-mer that reduces HSC activation by eliminating excess activated SECs.

In conclusion, hepatic PEDF is an intrinsic antifibrotic factor; however, its amount is reduced dramatically in the fibrotic liver. Here, we provide evidence supporting the hypothesis that the antifibrotic activity of PEDF is preserved in its 34-mer motif. The antifibrotic effect of the 34-mer was confirmed in an *in vivo* mouse model of CCl_4_-induced hepatic fibrosis and the antiproliferative and antifibrotic effects of the 34-mer on activated HSCs were validated in the *in vitro* study. The results of this study suggest that the PEDF-derived short peptide(s) may be used as an antifibrotic agent for treating liver fibrosis.

## Supporting Information

Figure S1 **The 34-mer prevents the accumulation of α-SMA and COL1A1 proteins in CCl_4_-treated mice.** Whole liver protein lysates at week 7 post-CCl_4_ treatment were extracted for western blot analysis with the indicated antibodies. Representative blots (A) and densitometric analysis (B) from three independent experiments are shown. *P<0.001 *versus* control peptide+CCl_4_-treated group.
(TIF)

Figure S2 **PPARγ antagonist abrogates the inhibitory effect of the 34-mer on PDGF signaling.** HSC-T6 cells were either untreated, treated with the 34-mer or co-treated with the 34-mer and PPARγ antagonist (GW9662 and G3335) for 2 days and then stimulated with PDGF for 5 and 10 min. Cells were harvested and subjected to western blot analysis with phosphospecific antibodies to ERK1/2 and Akt. Equal protein loading was confirmed by the reprobing the membranes with total ERK and Akt antibodies. Representative blots from three separate experiments are shown.
(TIF)
