# Correction: Pigment Epithelium-Derived Factor 34-mer Peptide Prevents Liver Fibrosis and Hepatic Stellate Cell Activation through Down-Regulation of the PDGF Receptor

**DOI:** 10.1371/journal.pone.0108835

**Published:** 2014-09-29

**Authors:** The PLOS ONE Staff

There is an error in affiliation 1 for authors Tung-Han Tsai, Hsin-I Ma, and Ming-Ying Liu. Affiliation 1 should be: Department of Neurosurgery, Tri-Service General Hospital, National Defense Medical Center, Taipei, Republic of China.
