# Supplementary figures and images for: Pigment Epithelium-Derived Factor 34-mer Peptide Prevents Liver Fibrosis and Hepatic Stellate Cell Activation through Down-Regulation of the PDGF Receptor
Source: PLoS One. 2014 Apr 24;9(4):e95443. doi: 10.1371/journal.pone.0095443 (PMC3998957; doi:10.1371/journal.pone.0095443)

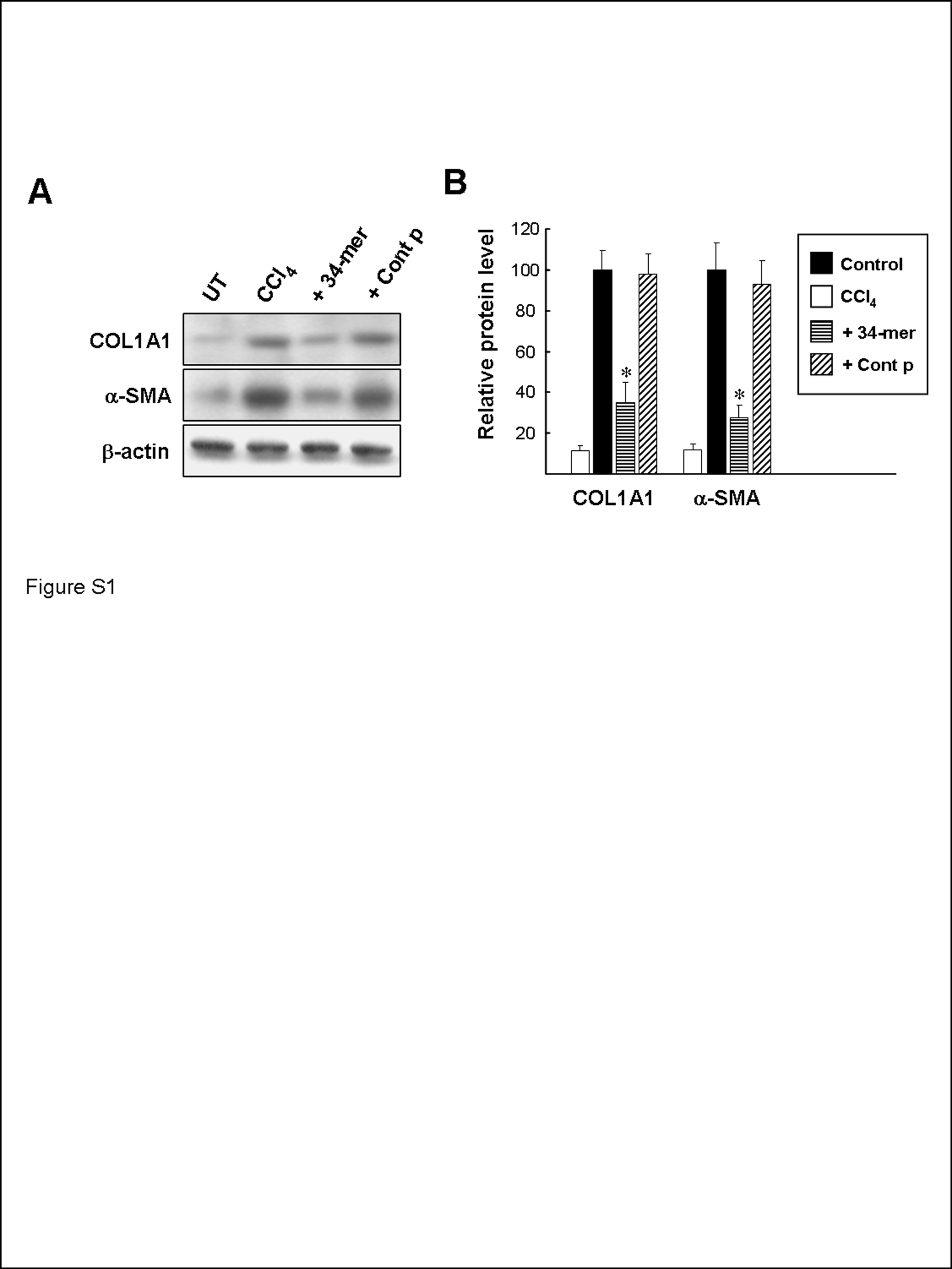

Supplement: Figure S1 — The 34-mer prevents the accumulation of α-SMA and COL1A1 proteins in CCl4-treated mice. Whole liver protein lysates at week 7 post-CCl4 treatment were extracted for western blot analysis with the indicated antibodies. Representative blots (A) and densitometric analysis (B) from three independent experiments are shown. *P<0.001 versus control peptide+CCl4-treated group. (TIF) [file pone.0095443.s001.tif]

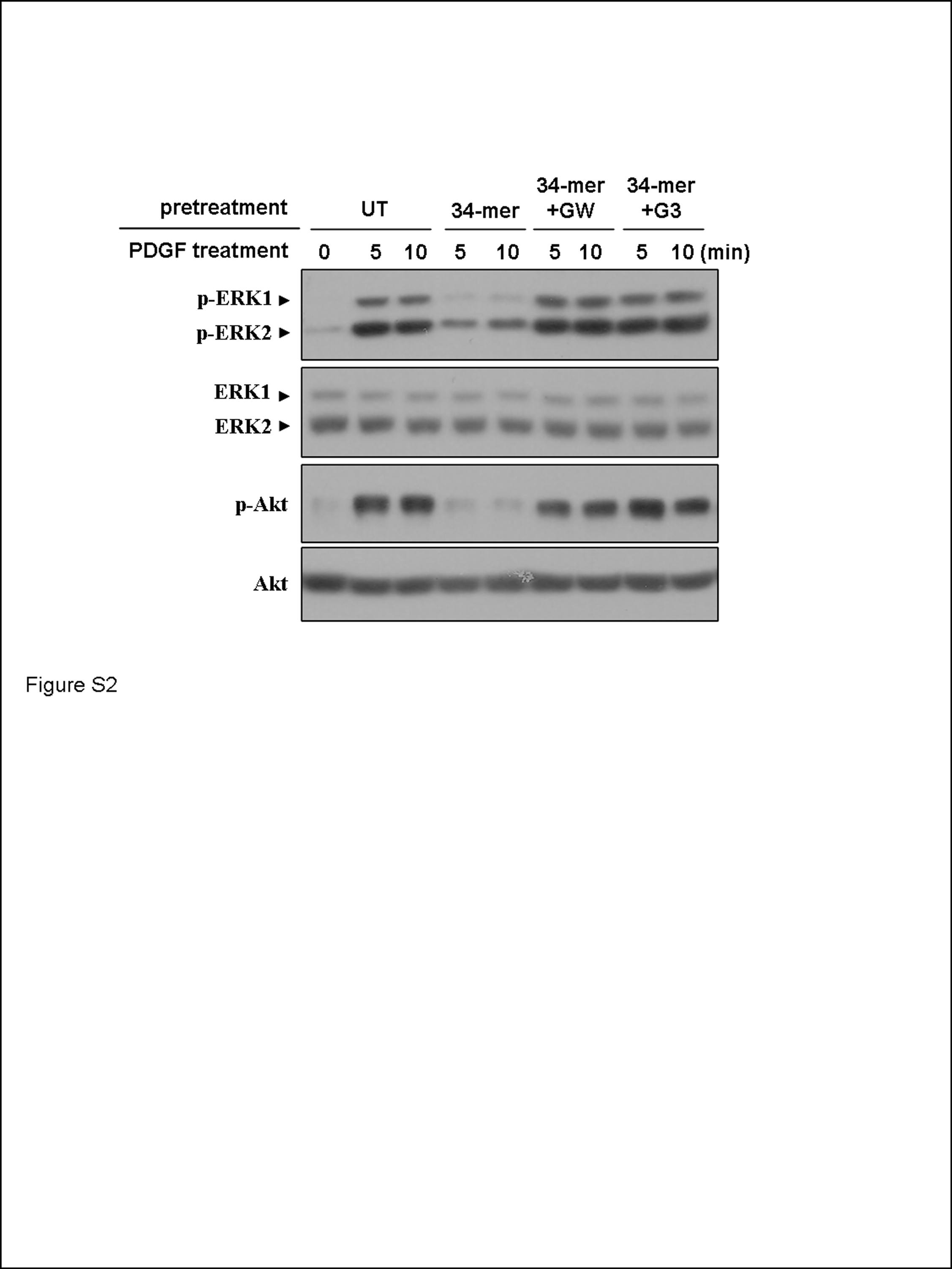

Supplement: Figure S2 — PPARγ antagonist abrogates the inhibitory effect of the 34-mer on PDGF signaling. HSC-T6 cells were either untreated, treated with the 34-mer or co-treated with the 34-mer and PPARγ antagonist (GW9662 and G3335) for 2 days and then stimulated with PDGF for 5 and 10 min. Cells were harvested and subjected to western blot analysis with phosphospecific antibodies to ERK1/2 and Akt. Equal protein loading was confirmed by the reprobing the membranes with total ERK and Akt antibodies. Representative blots from three separate experiments are shown. (TIF) [file pone.0095443.s002.tif]
